# Transforming Surgery With Artificial Intelligence: An Early Analysis of Private Industry Trends

**DOI:** 10.7759/cureus.82328

**Published:** 2025-04-15

**Authors:** Yash B Shah, Akshay S Krishnan, Zachary N Goldberg, Varun Jayanti, Erika D Harness, David B Nash

**Affiliations:** 1 Sidney Kimmel Medical College, Thomas Jefferson University, Philadelphia, USA; 2 College of Population Health, Thomas Jefferson University, Philadelphia, USA

**Keywords:** artificial intelligence, healthcare management, innovation, outcomes, surgery

## Abstract

Introduction: The recent growth and integration of artificial intelligence (AI) into medical products have the potential to revolutionize surgical care. The private sector is largely responsible for this innovation. We aimed to characterize the growth, aims, and finances of private industry AI solutions within surgery.

Methods: An initial search using the CB Insights market intelligence platform returned 126 private companies; 20 met the exclusion criteria. Three independent reviewers extracted variables of interest, including company demographics, product classification, surgical subspecialty of interest, funding, valuation, and acquisition status. Product purpose and functionality were characterized in detail.

Results: The first company was founded in 2003, with 50% (n=53) founded between 2015 and 2019. General surgery (n=57) and orthopedics (n=21) were the most common surgical subspecialties. The most common product categories were intraoperative image analysis (n=25), aiming to improve the visualization or navigation of the surgical site, and diagnostic imaging (n=18), aiming to improve the efficiency and accuracy of diagnosis and surgical planning. Of note, 12 companies aimed to increase the autonomy of surgical robotics. Finances were notably right-skewed, and total industry valuation exceeded one billion dollars, while six companies had been acquired.

Conclusions: There are numerous companies implementing AI solutions, and financial investment is high. Given the rapid pace of development, surgeon input is needed to reconcile product development with patient-centered care. Clinician participation in private industry development may address health inequities, shape the future role of surgeons in the operating room, and ensure safeguards with regulatory oversight and improved data reporting.

## Introduction

Attention surrounding the integration of artificial intelligence (AI) into medicine has dramatically increased in recent years [1]. While no literature has published the percent increase in AI use, a significant amount of literature about AI use in healthcare has been published. For example, one study found 43,485 articles on AI in elderly healthcare [2]. AI may increase diagnostic validity and patient safety, predict therapeutic response, and even accelerate clinical research processes [3-6]. Initial forays in the late 20th century involved simple AI tools that supported clinician decision-making by providing recommendations using predefined rules and a defined fund of medical knowledge (e.g., MYCIN); this has since evolved with the advent of big data analytics and deep learning (DL) algorithms [7].

Scalable devices like wearables have increased private sector engagement in population health data collection and research [8]. For example, the Apple Heart Study, which began in 2018, compared the accuracy of Apple Watch data against ambulatory electrocardiogram (ECG) in identifying atrial fibrillation, finding an 84% positive predictive value [9]. These technologies reflect the potential of generative AI, a subset of DL.

AI has also been incorporated into surgical care, supporting the allocation of post-anesthesia beds, prediction of case cancellations, enhancement of robotic movements and visibility, and creation of written materials such as patient education or clinical documents [10-12]. Surgical process analysis has been facilitated by breaking down individual surgical movements into "surgemes" (e.g., pulling a needle through tissue or transferring a needle from one hand to the other), which allows the computerized analysis, replication, and understanding of surgical tasks [13].

While academic and public sector research has laid the groundwork for AI in surgery, it is the private industry that has propelled these innovations into real-world applications [14]. For example, an investigation into the funding of 41 biopharmaceutical agents that received Food and Drug Administration (FDA) approval found that 97.4% of funding came from the private sector [15]. AI giants like NVIDIA and established healthcare companies like Johnson & Johnson have collaborated on innovative technologies in surgical virtual reality (VR) and education. This emerging industry appears poised to expand its impact across surgical specialties and will have significant impacts on the role of surgeons in the coming decades. Our study aimed to analyze private sector companies offering surgical AI products.

## Materials and methods

CB Insights (CBI) database

The CBI market intelligence platform gathers data about private companies and investments to track market trends across a variety of sectors. This tool has been previously utilized to analyze private industry investments in healthcare [16-18]. The present study utilized the Artificial Intelligence Expert Collection, which contained 13,607 listings. The Healthcare & Life Sciences Industry categorization was utilized to ultimately identify private companies for inclusion.

Search and cohort identification

The search was conducted on March 10, 2024. The Boolean keyword search "artificial intelligence AND surgery" was utilized to filter the original database. The initial search yielded 126 results. Following the identification of this cohort, three independent reviewers viewed the company URLs provided by CBI. Further, all reviewers utilized Google Search to investigate each listing further. This investigation served as a check on CBI to ensure the selected companies met the inclusion criteria. Exclusion criteria included duplicates, inactive companies, and those that did not create surgical products involving AI. Companies were determined to involve AI or not based on the centrality of AI to their product, such as considering if the product was built around AI or if the product could function the same without AI integration. Disagreements between reviewers were discussed until a consensus was reached.

Data analysis

Variables of interest included the company's founding date, location of headquarters, total funding in 2024 US dollars (USD), most recent valuation, and most recent acquisition, if applicable. Further, the company's mission, proposed product, market status of product, surgical subspecialty of interest, and classification of product type were gathered. Information was gathered from CBI profiles, the company's website, and supporting articles from Google Search in that order. Data on collaboration with academic institutions, National Institutes of Health (NIH) funding, or other grant support was unavailable.

If a company fit into general surgery or multiple specialties or involved generic surgical tasks, it was categorized as "General". Product classifications included surgical support through intraoperative image analysis (including surgical microscopy, recording of procedures, and endoscopic or robotic video output), diagnostic imaging, diagnostic pathology, operating room (OR) workflow management, robotic surgical support (control of surgical robots), physician education through simulation and training, physician education through preoperative surgical preparation, routine physician tasks and paperwork, patient navigation, postoperative monitoring software, and postoperative monitoring devices.

Market status was classified as "Active" if the product was currently being sold or had been approved by the FDA or equivalent regulatory body for sale, while it was classified as "Under Development" if the company was in the seed funding, research and development, or early product trial phases at select healthcare sites.

## Results

Company demographics

The CBI search returned 126 companies, of which 17 (13.5%) were excluded from the study due to inactivity (11), lack of surgical uses (four), or lack of AI incorporation (two). An additional three companies (2.4%) were eliminated for being duplicates. In total, 106 private companies creating surgical products using AI were included in the analysis (Table 1). Companies were headquartered on all inhabited continents. The United States had the most companies (n=42), followed by China (n=14). France, India, and the United Kingdom each had six companies, and South Korea had five companies. No other country had more than three companies.

**Table 1:** Index of companies creating surgical products with AI AI: artificial intelligence; AR: augmented reality; CT: computed tomography; DL: deep learning; EHR: electronic health record; ML: machine learning; MRI: magnetic resonance imaging; OR: operating room; Post-op: postoperative; VR: virtual reality

Companies	Category	Product	Specialty	Country	Year founded
True Digital Surgery	Surgical support through intraoperative image analysis	AI-powered tool allowing advanced surgical microscopy	General	United States	2003
Hongyun Rongtong	Surgical support through intraoperative image analysis	AI-powered provider-provider telecommunication for remote intraoperative consultation	General	China	2008
Shareable Forms	Routine physician tasks/paperwork	Enterprise EHR analytics for peri- and intraoperative patient monitoring	General	United States	2009
XSurgical Robotics	Robotic surgical support	AI-powered robot to assist and perform surgeries	General	Italy	2009
Sensoria	Post-op monitoring device	AI-powered wearable devices to track movement post-surgery	Orthopedic	United States	2010
Gauss Surgical	Surgical support through intraoperative image analysis	AI-powered tracking of real-time blood loss via analysis of video footage	General	United States	2011
DeGen Medical	Physician education through surgical prep	AI/AR platform to help surgeons plan and practice spinal surgeries	Orthopedic	United States	2011
Avamed Synergy	Surgical support through intraoperative image analysis	AI-powered intraoperative image analysis to improve outcomes	General	Spain	2012
nView medical	Diagnostic imaging	AI image recognition for diagnostic assistance pre- and post-op	Neurosurgery	United States	2012
Digital Surgery	Physician education through simulation and training	AI-powered mobile app providing surgical training and simulation	General	United Kingdom	2013
Caresyntax	OR workflow management	Platform enabling data-driven surgery to reduce variability and improve efficiency	General	Germany	2013
Neo	Surgical support through intraoperative image analysis	AI/AR platform to improve visualization during spinal surgery	Neurosurgery	Switzerland	2013
Fundamental Surgery	Physician education through simulation and training	AI-/VR-powered surgical training platform	General	United Kingdom	2013
DocSpera	OR workflow management	Enterprise solution for clinical workflow and surgical resource management	General	United States	2013
Deshang Yunxing	Diagnostic imaging	AI image recognition for diagnostic assistance, surgery planning, and intraoperative surgical guidance	General	China	2013
Perceive3D	Surgical support through intraoperative image analysis	AR platform to improve intraoperative visualization	Orthopedic	Portugal	2013
Zhongzhi Daxin	Physician education through simulation and training	AI-powered tools for surgical planning, online collaboration, and 3D printing	General	China	2014
Cydar Medical	OR workflow management	Enterprise solution for clinical workflow and surgical resource management	Cardiovascular	United Kingdom	2014
Axial3D	Diagnostic imaging	AI-powered platform to make virtual and 3D printed models for surgical planning	Orthopedic	United Kingdom	2014
Vibronix	Diagnostic imaging	AI image recognition for diagnostic assistance, surgery planning, and intraoperative surgical guidance	General	United States	2014
PeerWell	Post-op monitoring device	AI-powered wearable device to improve preoperative patient readiness	Orthopedic	United States	2015
Saykara	Routine physician tasks/paperwork	AI-powered analysis of appointment audio for automatic charting	General	United States	2015
Reveal Surgical	Diagnostic pathology	AI-assisted intraoperative visualization and cancer detection tool	Neurosurgery	Canada	2015
ME3D	Physician education through simulation and training	AI/AR tool to train surgeons how to stitch blood vessels	General	Hungary	2015
Oxford Heartbeat	Physician education through surgical prep	AI-powered platform to make virtual and 3D printed models for surgical planning	Neurosurgery	United Kingdom	2015
PTX Therapy	Post-op monitoring software	AI-powered personalized physical therapy course	General	United States	2015
Unify Medical	Surgical support through intraoperative image analysis	AI/AR platform to improve visualization through 3D models and AI-enhanced magnification	General	United States	2015
OrthoGrid Systems	Surgical support through intraoperative image analysis	AI-powered intraoperative surgical assistance using fluoroscopy data	Orthopedic	United States	2015
AUTONOMUS	Robotic surgical support	AI-powered robot to perform ultrasound-guided surgeries	General	United States	2015
Keya Medical	Diagnostic imaging	AI-powered CT angiograph reader to assess coronary artery function	Cardiovascular	China	2016
BossCome	Surgical support through intraoperative image analysis	AI-powered tool to improve image-guided surgeries	General	China	2016
TriOcula	Physician education through simulation and training	AI-/AR-/VR-powered surgical training platform	General	India	2016
Formus Labs	Diagnostic imaging	AI-powered platform to make virtual and 3D printed models for surgical planning and implantation	Orthopedic	New Zealand	2016
Ceevra	Diagnostic imaging	AI-powered platform to make 3D printed models for surgical planning	General	United States	2016
Proprio	Surgical support through intraoperative image analysis	AR/VR technology providing immersive surgical vision	Orthopedic	United States	2016
CognitiveDOC	Post-op monitoring software	AI-powered app to reduce readmission	Orthopedic	United States	2016
UpFlux	OR workflow management	Enterprise solution for clinical workflow and surgical resource management	General	Brazil	2017
IHS	Physician education through simulation and training	VR-/AI-powered surgical training platform using haptic simulation	General	China	2017
Changmugu Medical	Diagnostic imaging	AI-powered platform to aid diagnostics, operative planning, and 3D printing design for custom bones and prosthetics	Orthopedic	China	2017
International Rehabilitation Institute	Post-op monitoring software	AI-powered postoperative rehabilitation monitoring	Orthopedic	China	2017
LaraLab	Physician education through surgical prep	AI-powered software for preprocedural surgical planning	Cardiovascular	Germany	2017
Healthplus.ai	Post-op monitoring software	AI-powered prediction of post-surgical complications	General	Netherlands	2017
Hutom	Surgical support through intraoperative image analysis	AI-powered platform providing preoperative anatomy modeling, surgical visualization, and detection of intraoperative events for intraoperative images	General	South Korea	2017
Activ Surgical	Surgical support through intraoperative image analysis	AI/AR/ML platform that enables real-time surgical landmarking	General	United States	2017
OpticSurg	Surgical support through intraoperative image analysis	AI-/AR-powered provider-provider telecommunication for remote intraoperative consultation	General	United States	2017
Pathware	Diagnostic pathology	AI-assisted intraoperative visualization and cancer detection tool	Oncology	United States	2017
Med Cloud Tech	Surgical support through intraoperative image analysis	AI-powered platform to improve visualization during screw placement	Orthopedic	United States	2017
Kaliber.ai	Surgical support through intraoperative image analysis	AI-powered suite of tools allowing patients and providers to analyze segments of the surgery	Orthopedic	United States	2017
InformAI	Diagnostic imaging	AI-powered predictive analytics using imaging and test results to predict treatment plans, transplant outcomes, and presence of disease	Transplant	United States	2017
Medicalfile	Routine physician tasks/paperwork	AI-powered EHR allowing for faster information entry	General	Mexico	2017
Scalpel	OR workflow management	AI-powered surgical inventory management	General	United Kingdom	2017
True Health	Diagnostic imaging	AI image recognition for diagnostic assistance pre- and post-op	General	China	2018
Ganymed Robotics	Robotic surgical support	AI-powered robot to assist with knee replacement and improve visualization	Orthopedic	France	2018
Theator	Physician education through simulation and training	AI-powered platform analyzing surgical recordings to train residents and increase efficiency and consistency	General	United States	2018
Zeta Surgical	Surgical support through intraoperative image analysis	AI-powered platform to improve image-guided surgeries	Neurosurgery	United States	2018
CytoVeris	Diagnostic pathology	AI-/ML-assisted intraoperative visualization and cancer detection	Oncology	United States	2018
Boea Wisdom	Physician education through surgical prep	AI-powered software using patient scans to assist with preoperative tasks and planning	Cardiovascular	China	2019
Yurui Innovation	Physician education through surgical prep	AI-powered surgical planning and postoperative feedback	General	China	2019
Caranx Medical	Robotic surgical support	AI-powered robot to assist with cardiac- and obesity-related surgeries	General	France	2019
Taurean Surgical	Surgical support through intraoperative image analysis	AI-enhanced surgical microscopy	General	India	2019
Rxoom	Surgical support through intraoperative image analysis	AI-powered intraoperative image analysis to improve neurosurgeon navigation	Neurosurgery	India	2019
iMed Technologies	Surgical support through intraoperative image analysis	AI/AR platform to improve visualization during neurovascular surgery	Neurosurgery	Japan	2019
Kyalio	Physician education through simulation and training	AI-/VR-powered surgical training platform	General	Singapore	2019
Pipra	Post-op monitoring software	AI-powered data-driven prediction of postoperative delirium	General	Switzerland	2019
CaseCTRL	OR workflow management	Enterprise solution for improved OR scheduling	General	United States	2019
NeuroVascular Research & Design	OR workflow management	AI-powered intraoperative documentation and monitoring	General	United States	2019
8chili	Physician education through simulation and training	AR/ML program for remote collaborative surgical training	General	United States	2019
Remedy Logic	Diagnostic imaging	AI-powered tool to increase MRI reading speeds	Neurosurgery	United States	2019
MS Pen Technologies	Diagnostic pathology	AI-assisted intraoperative visualization and cancer detection tool	Oncology	United States	2019
Caira	Robotic surgical support	AI-powered robot to assist with implant positioning and kinematics	Orthopedic	United States	2019
SurgAR	Surgical support through intraoperative image analysis	AR platform to improve visualization and intraoperative decision-making	General	France	2019
AIkenist	Diagnostic imaging	AI-powered suite of tools streamlining the entire imaging process	General	India	2019
Ortoma	Physician education through surgical prep	AI-assisted peri- and postoperative patient monitoring	Orthopedic	Sweden	2019
Skinopathy	Diagnostic imaging	AI image recognition for skin cancer screening and monitoring	Dermatology	Canada	2020
Oculotronics	Robotic surgical support	AI-powered robots to perform ophthalmic surgery	Orthopedic	China	2020
Alviss.ai	Diagnostic imaging	AI-powered predictive analytics using CT scans to predict the risk of complications like pulmonary embolism	Cardiovascular	France	2020
Anaut	Surgical support through intraoperative image analysis	AI-/DL-powered mapping of surgical images for improved navigation	General	Japan	2020
Rootally AI	Post-op monitoring software	AI-powered personalized physical therapy and wellness course	General	Singapore	2020
NATOO	Patient navigation	AI-powered tool for patients to choose their plastic surgery options	Plastics and Cosmetics	South Korea	2020
Apella	OR workflow management	Enterprise solution for clinical staff allocation	General	United States	2020
ORtelligence	OR workflow management	Enterprise solution for clinical workflow and surgical resource management	General	United States	2020
Opollo Technologies	OR workflow management	Enterprise solution for clinical workflow and surgical resource management	General	United States	2020
Cyberdontics	Robotic surgical support	AI-powered robot to perform dental procedures	Plastics and Cosmetics	United States	2020
Saude iD	Patient navigation	AI-powered digital marketplace for patients seeking surgery	General	Brazil	2020
IRCAD	Physician education through simulation and training	AI-powered surgical training platform and novel surgical technique development	General	France	2020
Your Physio	Post-op monitoring software	AI-powered personalized physical therapy course	Orthopedic	India	2020
Nervio	Surgical support through intraoperative image analysis	AI-powered surgeon monitors to stop complications before they happen	Neurosurgery	Israel	2020
QAS.AI	Surgical support through intraoperative image analysis	AI-powered intraoperative angiogram and perfusion analysis	Neurosurgery	United States	2020
Oma Robotics	Robotic surgical support	AI-powered robot to perform single cell surgery	Fertility	United States	2020
Vope Medical	Surgical support through intraoperative image analysis	AI-powered software for endoscope cleaning	General	Canada	2021
AMIT	Robotic surgical support	AI-powered robot to perform ultrasound-guided ablations	General	China	2021
AIMIRA	Robotic surgical support	AI-powered robot to perform cosmetic procedures	Plastics and Cosmetics	China	2021
TrackiMed	Robotic surgical support	AI-powered robot to manage the OR and assist with procedures	General	Israel	2021
Proid	Surgical support through intraoperative image analysis	AI- and 3D optics-powered platform to improve tumor visualization	Oncology	South Korea	2021
Connecteve	Diagnostic imaging	AI-powered software to assist with pre- and postoperative tasks and planning	Orthopedic	South Korea	2021
BiteRight	Diagnostic imaging	AI-powered platform to make custom dental implants	Plastics and Cosmetics	Spain	2021
Sterile Vision	OR workflow management	AI-powered surgical inventory management	General	United States	2021
Innobot Health	Routine physician tasks/paperwork	AI-powered automation of eligibility, authorization, appeal, record request, and payment forms	General	United States	2021
Bonescreen	Diagnostic imaging	AI image and biomarker recognition for diagnostic assistance	Orthopedic	Germany	2022
INFINEIS	Diagnostic imaging	AI-powered tool to make custom surgical implants	General	France	2022
Scichip	Robotic surgical support	AI-powered robot and software to plan and perform laparoscopic surgeries	General	India	2022
Med4cast	Post-op monitoring software	AI-powered data-driven prediction of postoperative outcomes	Orthopedic	Switzerland	2022
Aivigo	Post-op monitoring software	AI-powered personalized physical therapy course	General	Turkey	2022
Oculotix	Physician education through surgical prep	AI-powered tool to help match patients with the correct lenses	Ophthalmology	United States	2022
W.AI	Post-op monitoring software	AI-powered software to monitor breast implants for complications	Plastics and Cosmetics	South Korea	2023
Exin Health	OR workflow management	Enterprise solution for clinical workflow and surgical resource management	General	United States	2023

The earliest company was founded in 2003, with less than 5% of companies being founded before 2010 (n=4). Fifty percent of the companies were founded between 2015 and 2019 (n=53), and 2019 was the year with the most companies founded (n=17) (Table 2). In total, 56 (52.8%) companies had products actively available in the market. 

**Table 2:** Distribution of companies by year founded

Year founded	n (%)
Before 2010	4 (3.8%)
2010-2014	16 (15.1%)
2015-2019	53 (50%)
2020-present	33 (31.1%)

Classification of products

Distribution by surgical subspecialty was also assessed. While most companies were classified as General (n=57), Orthopedics (n=21) was also highly common, followed by Neurosurgery (n=10), Plastics and Cosmetics (n=5), Cardiovascular (n=5), and Oncology (n=4). Dermatology, Fertility, Ophthalmology, and Transplant each had one company.

The type of product or service provided was further categorized (Table 3). A plurality of products offered surgical support through intraoperative image analysis (n=25). These can improve intraoperative visualization and navigation of the surgical site. Additional uses included enhanced surgical microscopy, tracking of blood loss, or identification of landmarks and structures such as tumors.

**Table 3:** Categorization of surgical AI products Post-op: postoperative; OR: operating room; AI: artificial intelligence

Category	n (%)	Description
Surgical support through intraoperative image analysis	25 (23.6%)	Analysis of images taken during a procedure to improve the surgeon's view of the surgical site
Diagnostic imaging	18 (17%)	Analysis of images taken outside of surgery to aid in diagnosis
OR workflow management	12 (11.3%)	Platforms which optimize the allocation of surgical resources including OR time, personnel time, and equipment
Robotic surgical support	12 (11.3%)	Robots to assist surgeons or perform surgeries alone
Post-op monitoring software	10 (9.4%)	Software designed to improve patient recovery after surgery
Physician education through simulation and training	10 (9.4%)	Simulation programs to help train residents on how to perform surgery
Physician education through surgical prep	7 (6.6%)	Programs to help surgeons practice and plan for specific surgical cases
Diagnostic pathology	4 (3.8%)	Devices that analyze a tissue sample to determine if it is cancerous or not in real time
Routine physician tasks/paperwork	4 (3.8%)	Products which automatically perform tasks so surgeons do not need to
Patient navigation	2 (1.9%)	Products which help patients navigate the surgical system
Postoperative monitoring devices	2 (1.9%)	Devices which allow care teams to monitor patients after surgery

The next most common product type was diagnostic imaging (n=18). These products analyze imaging taken before surgery to aid in diagnosis and surgical planning or the creation of 3D models for operative planning, surgical implant design, or risk prediction.

OR workflow management and robotic surgical support were the third most common product type (n=12). The former optimizes the flow of the OR through improved resource allocation or tracking of surgical resources, ultimately aiming to reduce cost, increase efficiency, and improve patient safety. Robotic surgical support products are designed to assist surgeons in performing procedures or complete procedures autonomously. These companies aim to increase standardization and safety of various surgeries while alleviating the burden on surgeons or improving efficiency.

Financial analysis

Of the 106 companies with surgical products using AI, six had been acquired at the time of data collection. These six companies came from General (n=4) and Orthopedics (n=2).

Fifty-six (52.8%) companies received venture capital investment. Fifty-five (51.9%) companies had funding information available. In total, this cohort of the industry saw $1.1 billion 2024 USD in funding. Of these, the mean funding was $19,550,000, while the median funding was $5,770,000. The company with the highest funding, Caresyntax, received $207,500,000, while the lowest, Vope Medical, received $200,000. The total industry valuation was $1.10 billion for the 19 companies with valuation data available. The mean valuation was $58,515,800, while the median valuation was $23,970,000. These funding values include money from private and government sources. Table 4 displays funding and Table 5 displays valuations for companies creating surgical products using AI. Table 6 displays funding and Table 7 displays valuations for all companies divided by where the company is headquartered.

**Table 4:** Distribution of company funding

Funding	n	Collective funding (millions)	% of total funding
Less than $5 million	23	$29.06	2.7%
$5-40 million	23	$273.17	25.4%
Greater than $40 million	9	$773.14	71.9%
Total	55	$1,075.37	100%

**Table 5:** Distribution of company valuations

Valuation	n	Collective valuation (millions)	% of total valuation
Less than $10 million	4	$23.85	2.2%
$10-24 million	6	$107.10	9.7%
$25-100 million	6	$357.21	32.4%
Greater than $100 million	3	$614.82	55.7%
Total	19	$1,102.98	100%

**Table 6:** Distribution of company funding by country

Country	n	Total funding (million)	% of total
Brazil	1	$9.25	0.9%
Canada	1	$0.02	0%
China	6	$232.78	21.6%
France	3	$45.45	4.2%
Germany	2	$208.98	19.4%
Japan	1	$1.61	0.1%
New Zealand	1	$5.75	0.5%
Portugal	1	$0.57	0.1%
Singapore	1	$0.88	0.1%
South Korea	5	$22.01	2%
Spain	1	$0.87	0.1%
Switzerland	3	$50.57	4.7%
United Kingdom	4	$70.13	6.5%
United States	25	$426.71	39.7%
Total	55	$1075.58	100%

**Table 7:** Distribution of company valuations by country

Country	n	Total valuation (million)	% of total
China	2	$17.4	1.6%
Germany	1	$366.23	33.2%
South Korea	2	$89.65	8.1%
United Kingdom	1	$14.14	1.3%
United States	13	$615.56	55.8%
Total	19	$1102.98	100%

## Discussion

Our study found that a wide range of companies, at various stages of development, are aiming to integrate advanced AI technologies into surgery. This trend is observed worldwide, though the most innovation is concentrated in the United States and China. This is expected as these countries are leading the front on innovation, technology, and medical science. While this trend has been seen for two decades, there has been increased growth in recent years. Funding is highly variable among companies, and the reasons for this variation are unclear. Possible explanations include differences in regulatory environments and confidence in company leadership. Further research is needed to explore the exact differences and their causes. Despite these differences, in total, this is a billion-dollar industry that bears notice. The billion-dollar valuation is likely an underestimate as around one-half of the studied companies lacked publicly available financial information or data that were unavailable in the CBI database. Further, it is likely that CBI was not a comprehensive database and missed relevant active companies.

Given the strong funding support and venture capital interest, it is reasonable to expect that AI integration into surgery will continue to expand. Therefore, it remains of interest to characterize this work and its potential to impact surgical patient experiences and the role of surgeons in the coming decades. Surgeons who are informed about this trend may positively guide new technologies to ensure patient-centered design and development.

Industry efforts are largely focused on facilitating the role of the surgeon to improve the efficiency and quality of care delivered [19]. The products offered by the companies in this study aim to simplify common tasks and overcome obstacles. For instance, intraoperative visualization and identification of key structures can be a routine challenge. Advanced technologies to facilitate this task can improve operative outcomes, such as reducing positive tumor margins or intraoperative bleeds, while yielding reduced operating times and mental burdens for the surgeon [19].

Proposed applications in surgical practice

Our study included 25 companies aiming to use OR video recording, surgical microscopy, or robotic video output to improve visualization for such purposes. Real-time assessment of bleeding using AI has been described in the literature and holds potential for reducing intraoperative morbidity [20]. One recent study showed the accurate detection of organs and surgical instruments using real-time gastrectomy video footage [21]. Similarly, effective cancer detection using video from endoscopy has been shown [22].

Assistance with preoperative diagnostics can alert physicians to key findings and help reduce complications, whether in missing an important finding or overtreating patients with lower-risk findings. AI use for spatial work in diagnostics, such as pathologic and radiologic analysis, has been demonstrated successfully [23]. In one study, a dataset of biopsy-proven high-risk breast lesions was used to train an algorithm to predict which patients needed surgical resection. The algorithm identified 30.6% of the resections performed as unnecessary [24]. In 2018, the FDA approved the first computer vision AI-driven medical device from LumineticsCore to identify diabetic retinopathy from patient images without the need for dilation [25].

Further, OR efficiency and safety culture oversight are areas ripe for technological improvement. AI-driven preoperative checklists and surgical time-out management can facilitate patient safety and reduce human error [26]. Utilizing AI to analyze surgical workflow and inventory, including staffing, scheduling, and the utilization of various materials and tools, can reduce waste and improve efficiency in an era of increasing costs and shortages.

Of further interest is the role of AI in training surgeons and assisting with preoperative planning [19]. The industry is heavily focused on improving the surgeon's ability to practice difficult procedures accurately and in high fidelity prior to treating real patients. This can ultimately improve surgeon comfort and skill while safeguarding the patient. As interest in simulation and VR rises, the integration of AI is promising [27].

Use cases

While this study focuses primarily on the economic and developmental trends surrounding AI integration in surgery, understanding the utility of these systems requires a look at how specific technologies are being used in practice. We, therefore, highlight several examples below to illustrate common applications.

Perhaps most exciting are the companies actively using AI within the OR. Activ Surgical has developed an AI-enabled imaging system for surgical landmarking, including real-time overlays of blood flow data on standard laparoscopic video. This assists surgeons in identifying poorly perfused tissue that may be at risk for complications. Proprio combines computer vision and mixed reality to create immersive visualizations of the surgical field, helping surgeons execute complex procedures with increased precision.

In the educational sphere, Theator analyzes video from past procedures to improve future performance, offering insights into technique variation and efficiency. Similarly, Kyalio has developed AI-driven training solutions, including performance analysis, structured feedback on technique, and remote collaboration with expert surgeons, particularly supporting practices in low-resource settings.

AI also holds increasing use in workflow management, reducing cost, turnover time, and resource utilization. CaseCTRL offers intelligent surgical planning by analyzing patient data and surgeon preferences to streamline case preparation and communication, minimizing last-minute changes and cancellations. ORtelligence focuses on logistics optimization by monitoring inventory, equipment usage, and workflow practices to reduce delays and waste. These diverse innovations demonstrate that AI is being developed not just for intraoperative assistance but also to improve coordination, resource management, and safety culture across the surgical care continuum.

These examples underscore the broader implications of AI in surgery, not only as a driver of investment and startup activity but as real tools with measurable impacts on surgical planning, visualization, and outcomes.

Risks of AI integration in surgery

The results of our study suggest that further research is needed to identify and minimize the serious risks of AI integration in surgical practice. It bears noting that 12 companies are proposing the integration of AI with surgical robots to reduce the role of the human surgeon in the OR. Image-guided procedures are particularly well-positioned to benefit from AI, which can automate decision-making and surgical movements [28]. It is essential to consider what role the human surgeon will be relegated to as they sit behind the console [29].

Importantly, device malfunctions are common, currently estimated at 75% of cases, and procedures must be designed to allow human surgeons to comfortably take over in case of complications [29]. Further, recent literature suggests that the need for humanism in patient interactions and the ability to determine when not to operate will remain the most vital characteristics of human surgeons [1].

Additional caution surrounds the potential for these technologies to exacerbate inequity and lack of access for rural or underserved populations. The infrastructure costs for information technology, data management, and technical support are immense [29]. Despite promises to democratize surgical education or patient access to world-class surgeons even in remote areas, these technologies may remain limited to well-funded and well-resourced academic institutions, possibly widening the gap [29].

Overall, the authors believe that there is an imminent need for a standardized approach to the assessment of new healthcare devices, including AI tools, similar to the rigorous approval process for pharmaceuticals [23]. A recent review of research on AI-enabled surgical decision support techniques found that scientific rigor and data reporting are suboptimal [30]. Without oversight, the accuracy and utility of these tools will remain unclear, and the determination of their safety for patients will be left up to individual physicians or institutions. Our analysis indicates that numerous such products are currently under design and will soon be available in practice.

Study limitations

This study has several limitations. Notably, while CBI offers the largest list of companies pursuing AI solutions in surgery, it is unclear whether this list is truly comprehensive [16-18]. Nonetheless, the authors are unaware of any other complementary database that may fill this gap. Similarly, several companies were missing financial data. Further, there is no publicly available stratified data on NIH funding or grants via academic collaborations. Though we have closely notated missing financial data in the results, there may be a bias in which companies had unavailable data. Further, there are a variety of definitions of AI, and our study may not have captured all relevant companies. Finally, the state of these products within the development and marketing cycle remains unclear, and we cannot fully ascertain how many of these products have seen wide uptake or real-world adoption. Further research into this industry is warranted to better understand its size and scope, the makeup of industry leadership and consultants, and implications for surgeons and their patients.

## Conclusions

The integration of AI into the surgical field, driven by private sector innovation, promises to transform aspects of daily practice. The present study highlights a global distribution of AI-driven healthcare companies and underscores a substantial financial interest in supporting these advancements. AI technologies are enhancing intraoperative methods, preoperative diagnostics and preparation, OR workflow, and surgical training, thereby promising to improve efficiency, safety, and patient outcomes. However, the need for assessment and oversight remains critical to ensure the safety and efficacy of these emerging technologies before widespread clinical adoption. These findings offer relevant thought points for practicing physicians, policymakers, and industry leaders.
